# Editorial: Advanced approaches in the diagnosis and treatment of diabetes mellitus and secondary complications

**DOI:** 10.3389/fendo.2023.1291637

**Published:** 2023-09-20

**Authors:** Ponnurengam Malliappan Sivakumar, Ram Prasad, Pranav Kumar Prabhakar

**Affiliations:** ^1^ Institute of Research and Development, Duy Tan University, Da Nang, Vietnam; ^2^ School of Medicine and Pharmacy, Duy Tan University, Da Nang, Vietnam; ^3^ Department of Ophthalmology and Visual Sciences, School of Medicine, The University of Alabama at Birmingham, Birmingham, Birmingham, AL, United States; ^4^ Research and Development Cell, Lovely Professional University, Phagwara, Punjab, India

**Keywords:** diabetes, diagnosis, secondary complication, retinopathy, nephropathy, diabetic foot (DF)

## Introduction

Diabetes mellitus, a chronic metabolic disorder, has reached epidemic proportions globally, posing significant challenges to healthcare systems worldwide. With the number of individuals affected by diabetes rising steadily, there is an urgent need for innovative approaches to improve the diagnosis and treatment of this complex condition. Furthermore, the prevention and management of secondary complications associated with diabetes are of paramount importance to enhance patients’ quality of life. In this editorial, we delve into the realm of advanced approaches that hold promise in tackling diabetes mellitus and its debilitating secondary complications. Accurate and timely diagnosis is crucial for effective management of diabetes mellitus. Emerging technologies, such as continuous glucose monitoring systems and point-of-care testing devices, have revolutionized diabetes diagnostics. These innovative tools provide real-time information on glucose levels, enabling individuals with diabetes to monitor their blood sugar levels more effectively. Additionally, advancements in genetic testing and biomarker analysis have the potential to enhance risk prediction and personalized treatment strategies for diabetes.

## Individualized treatment strategies

Diabetes management is no longer a one-size-fits-all approach. Tailored treatment plans based on individual characteristics, including genetics, lifestyle factors, and comorbidities, are essential for optimizing patient outcomes. Recent developments in the field of pharmacogenomics have shed light on genetic variations that influence an individual’s response to specific antidiabetic medications. This knowledge allows for personalized medication selection, minimizing adverse effects and improving therapeutic efficacy. Moreover, the integration of digital health technologies, such as mobile applications and wearable devices, empowers patients to actively participate in their treatment, promoting self-management and adherence to treatment regimens. Addressing the myriad of secondary complications associated with diabetes requires a comprehensive and multidisciplinary approach. Collaborative efforts among healthcare professionals, including endocrinologists, cardiologists, ophthalmologists, nephrologists, and dietitians, are crucial in preventing and managing complications such as cardiovascular diseases, retinopathy, nephropathy, and neuropathy. The advent of telemedicine and telehealth services has further facilitated interdisciplinary collaboration, enabling remote patient monitoring, consultations, and timely interventions.

## Regenerative medicine and novel therapies

Regenerative medicine, with its potential to restore or replace damaged pancreatic beta cells, holds immense promise for individuals with diabetes. Stem cell therapy, tissue engineering, and gene therapy are among the innovative approaches being explored to regenerate insulin-producing cells and restore normal glucose metabolism. Furthermore, advancements in targeted drug delivery systems and nanomedicine offer exciting possibilities for more efficient and precise drug delivery, reducing systemic side effects and enhancing treatment efficacy. Preventing the onset of diabetes and its complications is an essential aspect of comprehensive diabetes care. Public health initiatives focusing on promoting healthy lifestyles, raising awareness, and implementing early screening programs are vital in curbing the diabetes epidemic. Innovative interventions such as digital health interventions, community-based programs, and educational campaigns can empower individuals to make informed decisions regarding diet, physical activity, and overall diabetes management.

The follow-up volume, which began publication in August 2022, focused primarily on areas of the secondary complications of diabetes and their management. It should address the treatment of diabetes, abnormalities of secondary complications, and other diseases associated with diabetes. The original volume’s focus was broad.

Submissions were solicited for the article types of Original Research (29), Review (5), Systematic Reviews (7), Case study (2), and Clinical trial (2), and special emphasis/invitation was promoted to the following topics: (1) Diabetic complications and its management; (2) Antibiotics and its involvement in diabetic complications; (3) Circadian rhythm and diabetic complications; (4) Performance of artificial intelligence in diabetic complications; (5) Post-operative complications in diabetic; (6) Hypertension (HTN) and diabetes mellitus type 2; (7) Association of epilepsy, anti-epileptic drugs (AEDs), and type 2 diabetes mellitus (T2DM); (8) Predictors of diabetes and prediabetes; (9) Nanomaterials for diabetic wound healing; (10) impact of oxidative stress-induced mitochondrial dysfunction on diabetic microvascular complications; (11) Case report: Diabetic muscle infarction with diabetic ketoacidosis.

Among the articles, the main focus was on the diagnosis and management of diabetes mellitus and secondary complications. As we know the main issue in the case of diabetes mellitus is secondary complications and it is the main reason for mortality and morbidity. Diabetic Kidney Disease is a prevalent and significant secondary complication often found in individuals with diabetes associated with retinopathies, nephropathies (Xie et al.), and renal failure, cardiovascular issues (Li et al.), and premature mortality. Over time, there has been a growing occurrence of normoalbuminuric diabetic kidney disease in both diabetic and diabetic kidney disease patients. This increase may be attributed to better diabetes management and potential changes in the kidney’s pathology in normoalbuminuric diabetic kidney disease (An et al.).

Examining the significance of circadian rhythms in the development of Type 2 Diabetes Mellitus (T2DM) involves assessing the interconnected metabolic processes, their association with circadian rhythms through both lifestyle and molecular angles, and their influence on the pathophysiology of T2DM. These effects have been substantiated through numerous research studies and have given rise to strategies such as time-restricted eating, chronotherapy (treatment timed according to circadian rhythms), and substances that stabilize circadian molecules (Hariri et al.).

The co-occurrence of HTN and T2DM was prevalent among the study participants of Haramaya University which forced us to put rigorous efforts into developing strategies for screening employees to tackle the alarming increase in HTN and T2DM in university employees (Motuma et al.). Chronic type 2 diabetes is linked to the disruption of collagen fiber organization, alterations in the extracellular matrix, and a decline in the biomechanical integrity of the rotator cuff tendon (Xu et al.). Elevated arterial rigidity is a prevalent occurrence among diabetes patients, and inflammation stands out as a primary factor contributing to this heightened arterial stiffness. Neutrophil-to-lymphocyte ratio outperformed platelet-to-lymphocyte ratio, and the combination of neutrophil-to-lymphocyte ratio and monocyte-to-lymphocyte ratio exhibited specific predictive capabilities signifying the rise in arterial stiffness among diabetes patients. These predictive values can facilitate the early detection of heightened arterial stiffness in individuals with diabetes (Ning et al.). Ultra-widefield color fundus photography along with high-speed ultra-widefield swept-source optical coherence tomography angiography showed strong concordance in identifying lesions associated with diabetic retinopathy and assessing its severity when compared to the combination of ultra-widefield color fundus photography and fluorescein angiography (Li et al.).

Diabetic kidney disease (DKD) is a prevalent chronic complication among individuals with diabetes, and its pathological diagnosis has significant shortcomings. Serum insulin-like growth factor-1 and interleukin-6 serve as biochemical markers for diagnosing and assessing the advancement of DKD and a combination of these two markers can enhance the sensitivity and specificity of the test (Liu et al.). The effect of a single urine C peptide/creatinine ratio can also be used in the estimation and assessment of islet β Cell function of T2DM patients with different renal functions (Zhou et al.). Antibiotic-infused bone cement demonstrates its effectiveness in the treatment of diabetic foot infection wounds, offering substantial savings in terms of medical resources and costs. Promoting its utilization in clinical practice is highly advisable. Moreover, to delve deeper into the topic of film formation induced by trauma bone cement in various stages of diabetic foot management, it is essential to conduct more multicenter trials with larger sample sizes in the future (Dong et al.).

## Case studies

One of the major consequences of diabetes that has a high death and disability rate is diabetic foot ulcer. Low-intensity ultrasound and blood microbubbles can improve the local soft tissue’s blood perfusion effect, which may help a diabetic ulcer heal more quickly (Zhang et al.). Diabetic muscle infarction, also known as diabetic myonecrosis, is a rare and long-term complication of poorly managed diabetes mellitus. It was discovered that acute diabetes decompensation, such as diabetic ketoacidosis, could also stimulate the occurrence and development of diabetic myonecrosis (Tang et al.).

## Clinical trial

According to recent research, the amniotic fluid includes significant amounts of multipotent mesenchymal, hematopoietic, neuronal, epithelial, and endothelial stem cells in addition to the elements necessary for wound repair. After 8-week studies on 92 type 2 diabetic patients between 2019-2022, it was established that the amniotic fluid represents a useful and safe option for treating chronic diabetic foot ulcers (Niami et al). The most prevalent chronic consequence of T2DM, distal symmetric polyneuropathy (DSPN), causes additional major problems such as diabetic foot ulcers, amputations, and decreased life expectancy. While interventional studies show that low-dose vitamin D treatment does not significantly ameliorate neuropathy in DSPN, observational studies suggest that vitamin D insufficiency may be linked to the development of DSPN in T2DM. Additionally, high-dose vitamin D supplementation is necessary for neuropathy healing to maintain optimum vitamin D levels (Chen et al.).

Peripheral nerve damage is a significant issue that disrupts neuronal communication, leading to a diminished quality of life and, in severe cases, permanent disability. Investigating tailored biomaterials, which mimic the extracellular matrix, holds promise for treating peripheral nerve injuries. These specialized biomaterials, owing to their positive biological effects, can accelerate tissue regeneration and act as vehicles for delivering cellular and pharmaceutical therapies. This opens up new avenues for using biomaterials to treat nerve damage (Raghav et al.).

## Conclusion

Outstanding improvements are being made in the detection, management, and prevention of diabetes mellitus and its consequences as a result of cutting-edge research and innovative technology. In order to improve patient outcomes and lessen the burden of this global health issue, it is critical that healthcare professionals, researchers, policymakers, and people with diabetes work together and take advantage of these breakthroughs. We can pave the way to a future in which diabetes mellitus and its secondary complications are successfully managed and ultimately prevented by adopting precision diagnosis, individual treatment strategies, multidisciplinary care, regenerative medicine, and a focus on prevention and early intervention.

## Author contributions

PS: Writing – review & editing. RP: Writing – review & editing. PP: Writing – original draft, Writing – review & editing.

